# Changes in Medical Schools

**Published:** 1860-09

**Authors:** 


PROFESSOR J. E. HOLBROOK has retired from the chair of Anatomy, in the Medical College of the State of North Carolina, and is succeeded by Dr. F. T. MILES, who is favorably known in Charleston as a private teacher of this branch.

